# 4-Meth­oxy­anilinium 2-carb­oxy-4,5-di­chloro­benzoate

**DOI:** 10.1107/S1600536813025014

**Published:** 2013-09-18

**Authors:** Graham Smith, Urs D. Wermuth

**Affiliations:** a Science and Engineering Faculty, Queensland University of Technology, GPO Box 2434, Brisbane, Queensland 4001, Australia

## Abstract

In the title salt C_7_H_10_NO^+^·C_8_H_3_Cl_2_O_4_^−^ the benzene rings of the cation and anion are essentially parallel [inter-ring dihedral angle 4.8 (2)°]. In the anion the carb­oxy­lic acid and carboxyl­ate groups make dihedral angles of 19.0 (2) and 79.5 (2)°, respectively, with the benzene ring. Aminium N—H⋯O, carb­oxy­lic acid O—H⋯O and weak aromatic C—H⋯O hydrogen-bonding associations with carboxyl O-atom acceptors together with cation–anion π–π ring inter­actions [minimum ring centroid separation = 3.734 (3) Å] give rise to a sheet structure lying parallel to (001).

## Related literature

For background to 4,5-di­chloro­phthalate salts, see: Mattes & Dorau (1986 ▶); Smith *et al.* (2008*a* ▶). For structures of some 1:1 anilinium salts of 4,5-di­chloro­phthalic acid, see: Odabaşoğlu & Büyükgüngör (2007 ▶); Smith *et al.* (2008*b* ▶); Smith *et al.* (2009 ▶). For the structure of a dianionic 4,5-di­chloro­phthalate salt, see: Smith & Wermuth (2012 ▶).
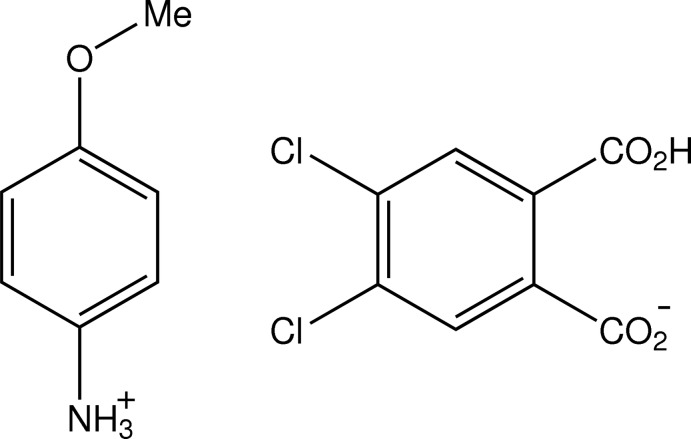


## Experimental

### 

#### Crystal data

C_7_H_10_NO^+^·C_8_H_3_Cl_2_O_4_^−^
*M* *_r_* = 358.16
Orthorhombic, 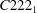

*a* = 7.5319 (8) Å
*b* = 12.9302 (14) Å
*c* = 32.3268 (18) Å
*V* = 3148.3 (5) Å^3^
*Z* = 8
Mo *K*α radiation
μ = 0.44 mm^−1^
*T* = 200 K
0.35 × 0.30 × 0.15 mm

#### Data collection

Oxford Diffraction Gemini-S CCD-detector diffractometer
Absorption correction: multi-scan (*CrysAlis PRO*, Agilent, 2012 ▶) *T*_min_ = 0.759, *T*_max_ = 0.980
6518 measured reflections
2730 independent reflections
2620 reflections with *I* > 2σ(*I*)
*R*_int_ = 0.036

#### Refinement

*R*[*F* ^2^ > 2σ(*F* ^2^)] = 0.067
*wR*(*F* ^2^) = 0.117
*S* = 1.42
2730 reflections
208 parameters
H-atom parameters constrained
Δρ_max_ = 0.32 e Å^−3^
Δρ_min_ = −0.33 e Å^−3^
Absolute structure: Flack (1983 ▶), 1569 Friedel pairs
Absolute structure parameter: 0.03 (13)

### 

Data collection: *CrysAlis PRO* (Agilent, 2012 ▶); cell refinement: *CrysAlis PRO*; data reduction: *CrysAlis PRO*; program(s) used to solve structure: *SIR92* (Altomare *et al.*, 1993 ▶); program(s) used to refine structure: *SHELXL97* (Sheldrick, 2008 ▶) within *ORTEP-3 for Windows* (Farrugia, 2012 ▶); molecular graphics: *PLATON* (Spek, 2009 ▶); software used to prepare material for publication: *PLATON*.

## Supplementary Material

Crystal structure: contains datablock(s) global, I. DOI: 10.1107/S1600536813025014/sj5350sup1.cif

Structure factors: contains datablock(s) I. DOI: 10.1107/S1600536813025014/sj5350Isup2.hkl

Supplementary material file. DOI: 10.1107/S1600536813025014/sj5350Isup3.cml

Additional supplementary materials:  crystallographic information; 3D view; checkCIF report

## Figures and Tables

**Table 1:** Hydrogen-bond geometry (Å, °)

*D*—H⋯*A*	*D*—H	H⋯*A*	*D*⋯*A*	*D*—H⋯*A*
N1*A*—H11*A*⋯O22^i^	0.81	2.10	2.881 (5)	163
N1*A*—H12*A*⋯O11^ii^	0.87	1.95	2.811 (5)	168
N1*A*—H13*A*⋯O12	0.99	1.84	2.814 (5)	167
O21—H21⋯O12^ii^	0.95	1.53	2.480 (4)	179
C3*A*—H3*A*⋯O12^iii^	0.95	2.50	3.265 (7)	137

Symmetry codes: (i) 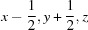; (ii) 
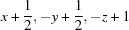
; (iii) 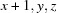.

**Fig. 1.**
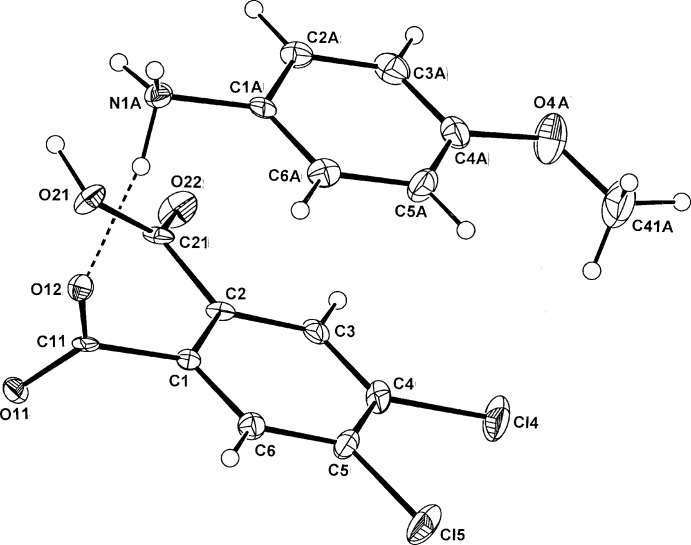
Molecular conformation and atom-numbering scheme for the cation and anion in the title salt, with the inter-species hydrogen bond shown as a dashed line. Non-H atoms are shown with 40% probability displacement ellipsoids.

**Fig. 2.**
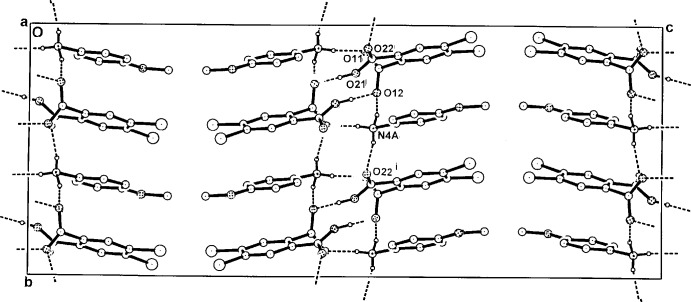
The two-dimensional structure in the unit cell viewed down the polymer layer, showing hydrogen-bonding associations as dashed lines. Non-associative H-atoms are omitted.

## supplementary crystallographic information

### 1. Comment

4,5-Dichlorophthalic acid (DCPA) most commonly forms 1:1 salts with organic Lewis bases, often giving low-dimensional hydrogen-bonded structures (Mattes & Dorau, 1986; Smith *et al.*, 2008*a*). The 1:2 DCPA salts are uncommon, as is the presence of water molecules of solvation, an example being the benzylamine salt (a monohydrate) (Smith & Wermuth, 2012). The salts with the aniline analogues are also not common, *e.g.* with 3-(trifluoromethyl)aniline (Odabaşoğlu & Büyükgüngör, 2007), the three isomeric carboxylanilines (Smith *et al.*, 2008*b* and 2-chloroaniline (Smith *et al.*, 2009). Our 1:1 stoichiometric reaction of DCPA with 4-methoxyaniline (*p*-anisidine) gave the anhydrous 1:1 salt C_7_H_10_NO^+^ C_8_H_3_Cl_2_O_4_^-^, the title compound, and the structure is reported herein.

In this structure (Fig. 1), the DCPA anion does not have the 'planar' conformation which is found in a large number of the 1:1 structures, but has the carboxylic acid and carboxylate groups rotated out of the benzene plane forming dihedral angles of 19.0 (2)° and 79.5 (2)°, respectively, with it. These correspond to torsion angles C1—C2—C21—O21 and C2—C1—C11—O11 of 19.1 (6) and 81.2 (5)°. In the crystal the cation and anion rings are close to parallel [inter-ring dihedral angle = 4.8 (2)°], giving layering along *b*. Weak π–π interactions are present between the benzene rings of the cations and anions [minimum ring centroid separation *Cg*···*Cg*^iv^ = 3.734 (3) Å] [for symmetry code (iv): *x* + 1/2, *y* + 1/2, *z*]. Aminium N—H···O, water O—H···O and weak aromatic C—H···O hydrogen-bonding associations with carboxyl O-atom acceptors (Table 1) give a two-dimensional sheet structure which lies parallel to (001) (Fig. 2).

### 2. Experimental

The title compound was synthesized by heating together for 10 min under reflux, 1 mmol quantities of 4,5-dichlorophthalic acid and 4-methoxyaniline (*p*-anisidine) in 50 ml of methanol. Partial evaporation of the solvent gave large colourless crystalline plates of the title compound (m.p. 473 K) from which a specimen was cleaved for the X-ray analysis..

### 3. Refinement

The aminium and carboxylic acid hydrogen atoms were located by difference methods but in the final refinement cycles these were allowed to ride on the parent atom, with *U*_iso_(H) = 1.2*U*_eq_(N) or 1.5*U*_eq_(O)]. Other H atoms were included at calculated positions [C—H (aromatic) = 0.95 Å or C—H (methyl) = 0.98 Å] and allowed to ride, with *U*_iso_(H) = 1.2 or 1.5*U*_eq_(C), respectively. Although not of great importance in this achiral molecule, the Flack parameter (Flack, 1983) was determined as 0.03 (13) for 1569 Friedel pairs. The TwinRotMat check [*PLATON* (Spek, 2009)] indicated no applicable twin law.

### Crystal data

**Table:**

C_7_H_10_NO^+^·C_8_H_3_Cl_2_O_4_^−^	*D*_x_ = 1.511 Mg m^−^^3^
*M**_r_* = 358.16	Melting point: 473 K
Orthorhombic, *C*222_1_	Mo *K*α radiation, λ = 0.71073 Å
Hall symbol: C 2c 2	Cell parameters from 2436 reflections
*a* = 7.5319 (8) Å	θ = 3.7–28.1°
*b* = 12.9302 (14) Å	µ = 0.44 mm^−^^1^
*c* = 32.3268 (18) Å	*T* = 200 K
*V* = 3148.3 (5) Å^3^	Plate, colourless
*Z* = 8	0.35 × 0.30 × 0.15 mm
*F*(000) = 1472	

### Data collection

**Table:**

Oxford Diffraction Gemini-S CCD-detector diffractometer	2730 independent reflections
Radiation source: Enhance (Mo) X-ray source	2620 reflections with *I* > 2σ(*I*)
Graphite monochromator	*R*_int_ = 0.036
Detector resolution: 16.077 pixels mm^-1^	θ_max_ = 25.0°, θ_min_ = 3.1°
ω scans	*h* = −8→8
Absorption correction: multi-scan (*CrysAlis PRO*, Agilent, 2012)	*k* = −15→16
*T*_min_ = 0.759, *T*_max_ = 0.980	*l* = −38→38
6518 measured reflections	

### Refinement

**Table:**

Refinement on *F*^2^	Secondary atom site location: difference Fourier map
Least-squares matrix: full	Hydrogen site location: inferred from neighbouring sites
*R*[*F*^2^ > 2σ(*F*^2^)] = 0.067	H-atom parameters constrained
*wR*(*F*^2^) = 0.117	*w* = 1/[σ^2^(*F*_o_^2^) + (0.0003*P*)^2^ + 10.2538*P*] where *P* = (*F*_o_^2^ + 2*F*_c_^2^)/3
*S* = 1.42	(Δ/σ)_max_ = 0.001
2730 reflections	Δρ_max_ = 0.32 e Å^−^^3^
208 parameters	Δρ_min_ = −0.33 e Å^−^^3^
0 restraints	Absolute structure: Flack (1983), 1569 Friedel pairs
Primary atom site location: structure-invariant direct methods	Absolute structure parameter: 0.03 (13)

### Special details

**Table:**

Geometry. Bond distances, angles *etc*. have been calculated using the rounded fractional coordinates. All su's are estimated from the variances of the (full) variance-covariance matrix. The cell e.s.d.'s are taken into account in the estimation of distances, angles and torsion angles
Refinement. Refinement of *F*^2^ against ALL reflections. The weighted *R*-factor *wR* and goodness of fit *S* are based on *F*^2^, conventional *R*-factors *R* are based on *F*, with *F* set to zero for negative *F*^2^. The threshold expression of *F*^2^ > σ(*F*^2^) is used only for calculating *R*-factors(gt) *etc*. and is not relevant to the choice of reflections for refinement. *R*-factors based on *F*^2^ are statistically about twice as large as those based on *F*, and *R*- factors based on ALL data will be even larger.

### Fractional atomic coordinates and isotropic or equivalent isotropic displacement parameters (Å^2^)

**Table:**

	*x*	*y*	*z*	*U*_iso_*/*U*_eq_	
Cl4	1.0614 (2)	0.04524 (14)	0.69649 (4)	0.0504 (5)	
Cl5	0.6536 (2)	0.09989 (13)	0.70946 (4)	0.0482 (5)	
O11	0.5402 (5)	0.1011 (3)	0.53015 (10)	0.0269 (11)	
O12	0.5633 (4)	0.2669 (2)	0.54919 (9)	0.0213 (10)	
O21	0.9047 (5)	0.1895 (3)	0.51574 (10)	0.0281 (11)	
O22	1.1354 (5)	0.0921 (3)	0.53458 (11)	0.0380 (12)	
C1	0.7212 (6)	0.1407 (3)	0.58796 (14)	0.0170 (14)	
C2	0.9001 (5)	0.1197 (3)	0.58236 (14)	0.0147 (12)	
C3	1.0023 (7)	0.0889 (4)	0.61632 (14)	0.0240 (16)	
C4	0.9274 (7)	0.0827 (4)	0.65531 (15)	0.0270 (17)	
C5	0.7505 (8)	0.1047 (4)	0.66078 (13)	0.0263 (16)	
C6	0.6454 (7)	0.1346 (4)	0.62745 (13)	0.0233 (16)	
C11	0.6000 (6)	0.1710 (4)	0.55224 (14)	0.0163 (12)	
C21	0.9909 (6)	0.1314 (3)	0.54121 (15)	0.0200 (16)	
O4A	1.2905 (6)	0.3233 (4)	0.67922 (14)	0.0607 (19)	
N1A	0.8469 (5)	0.4080 (3)	0.54416 (11)	0.0233 (11)	
C1A	0.9599 (6)	0.3876 (3)	0.58068 (14)	0.0203 (14)	
C2A	1.1396 (7)	0.3733 (4)	0.57540 (15)	0.0270 (17)	
C3A	1.2440 (8)	0.3499 (4)	0.60887 (18)	0.0350 (17)	
C4A	1.1695 (8)	0.3463 (4)	0.64817 (18)	0.0333 (19)	
C5A	0.9921 (8)	0.3605 (5)	0.65369 (16)	0.037 (2)	
C6A	0.8838 (7)	0.3818 (4)	0.61917 (15)	0.0270 (17)	
C41A	1.2233 (11)	0.3165 (6)	0.71999 (19)	0.072 (3)	
H3	1.12410	0.07220	0.61260	0.0290*	
H6	0.52360	0.15070	0.63140	0.0280*	
H21	0.96620	0.20640	0.49100	0.0420*	
H2A	1.19110	0.37960	0.54870	0.0320*	
H3A	1.36690	0.33630	0.60520	0.0420*	
H5A	0.94160	0.35610	0.68060	0.0450*	
H6A	0.75970	0.39200	0.62250	0.0330*	
H11A	0.80350	0.46520	0.54470	0.0280*	
H12A	0.89410	0.40030	0.51980	0.0280*	
H13A	0.75460	0.35580	0.54990	0.0280*	
H41A	1.32010	0.29960	0.73910	0.1080*	
H42A	1.13250	0.26230	0.72130	0.1080*	
H43A	1.17060	0.38290	0.72790	0.1080*	

### Atomic displacement parameters (Å^2^)

**Table:**

	*U*^11^	*U*^22^	*U*^33^	*U*^12^	*U*^13^	*U*^23^
Cl4	0.0580 (10)	0.0630 (11)	0.0301 (7)	0.0060 (9)	−0.0209 (7)	0.0088 (7)
Cl5	0.0724 (11)	0.0539 (10)	0.0182 (6)	0.0038 (9)	0.0077 (7)	0.0036 (7)
O11	0.031 (2)	0.0218 (18)	0.0280 (17)	−0.0087 (16)	−0.0101 (16)	0.0035 (16)
O12	0.0197 (18)	0.0207 (18)	0.0236 (16)	0.0035 (15)	−0.0036 (15)	0.0038 (14)
O21	0.027 (2)	0.036 (2)	0.0213 (17)	0.0074 (17)	0.0072 (15)	0.0107 (15)
O22	0.030 (2)	0.044 (2)	0.040 (2)	0.017 (2)	0.0067 (18)	0.0123 (19)
C1	0.026 (3)	0.005 (2)	0.020 (2)	−0.0021 (19)	−0.003 (2)	0.0001 (19)
C2	0.010 (2)	0.006 (2)	0.028 (2)	0.0044 (18)	0.0030 (19)	0.0034 (18)
C3	0.028 (3)	0.014 (2)	0.030 (3)	−0.006 (2)	−0.008 (2)	0.001 (2)
C4	0.038 (3)	0.014 (3)	0.029 (3)	0.006 (2)	−0.016 (3)	0.003 (2)
C5	0.041 (3)	0.022 (3)	0.016 (2)	0.000 (3)	−0.002 (2)	0.002 (2)
C6	0.024 (3)	0.022 (3)	0.024 (2)	0.000 (2)	−0.001 (2)	0.000 (2)
C11	0.007 (2)	0.019 (2)	0.023 (2)	−0.0040 (19)	0.0051 (18)	0.005 (2)
C21	0.016 (3)	0.011 (2)	0.033 (3)	−0.003 (2)	0.002 (2)	0.006 (2)
O4A	0.061 (3)	0.075 (4)	0.046 (3)	0.013 (3)	−0.024 (2)	0.010 (2)
N1A	0.027 (2)	0.020 (2)	0.0228 (19)	−0.0015 (19)	0.0007 (18)	0.0026 (18)
C1A	0.028 (3)	0.007 (2)	0.026 (2)	−0.006 (2)	−0.003 (2)	−0.001 (2)
C2A	0.022 (3)	0.026 (3)	0.033 (3)	−0.001 (2)	0.003 (2)	0.002 (2)
C3A	0.030 (3)	0.027 (3)	0.048 (3)	−0.001 (3)	−0.004 (3)	0.000 (3)
C4A	0.039 (4)	0.019 (3)	0.042 (3)	−0.003 (3)	−0.018 (3)	0.004 (2)
C5A	0.054 (4)	0.038 (4)	0.020 (3)	−0.004 (3)	0.002 (2)	0.002 (2)
C6A	0.033 (3)	0.016 (3)	0.032 (3)	0.003 (2)	0.004 (2)	−0.002 (2)
C41A	0.108 (7)	0.065 (5)	0.043 (4)	−0.007 (5)	−0.030 (4)	0.016 (4)

### Geometric parameters (Å, º)

**Table:**

Cl4—C4	1.739 (5)	C3—C4	1.383 (7)
Cl5—C5	1.736 (5)	C4—C5	1.374 (8)
O11—C11	1.237 (6)	C5—C6	1.392 (7)
O12—C11	1.274 (6)	C3—H3	0.9500
O21—C21	1.290 (6)	C6—H6	0.9500
O22—C21	1.220 (6)	C1A—C6A	1.372 (7)
O21—H21	0.9500	C1A—C2A	1.377 (7)
O4A—C41A	1.415 (8)	C2A—C3A	1.371 (8)
O4A—C4A	1.388 (7)	C3A—C4A	1.390 (8)
N1A—C1A	1.479 (6)	C4A—C5A	1.361 (9)
N1A—H13A	0.9900	C5A—C6A	1.409 (7)
N1A—H11A	0.8100	C2A—H2A	0.9500
N1A—H12A	0.8700	C3A—H3A	0.9500
C1—C11	1.523 (6)	C5A—H5A	0.9500
C1—C6	1.401 (6)	C6A—H6A	0.9500
C1—C2	1.386 (6)	C41A—H41A	0.9800
C2—C3	1.399 (6)	C41A—H42A	0.9800
C2—C21	1.503 (6)	C41A—H43A	0.9800
			
C21—O21—H21	115.00	C2—C3—H3	120.00
C4A—O4A—C41A	116.9 (5)	C4—C3—H3	120.00
H11A—N1A—H12A	107.00	C1—C6—H6	120.00
C1A—N1A—H13A	98.00	C5—C6—H6	120.00
H12A—N1A—H13A	112.00	C2A—C1A—C6A	121.1 (4)
H11A—N1A—H13A	110.00	N1A—C1A—C6A	119.6 (4)
C1A—N1A—H12A	118.00	N1A—C1A—C2A	119.4 (4)
C1A—N1A—H11A	112.00	C1A—C2A—C3A	119.7 (5)
C2—C1—C11	122.3 (4)	C2A—C3A—C4A	119.8 (5)
C6—C1—C11	117.5 (4)	O4A—C4A—C3A	113.8 (5)
C2—C1—C6	120.3 (4)	O4A—C4A—C5A	125.4 (5)
C1—C2—C21	122.5 (4)	C3A—C4A—C5A	120.8 (5)
C1—C2—C3	119.2 (4)	C4A—C5A—C6A	119.4 (5)
C3—C2—C21	118.2 (4)	C1A—C6A—C5A	119.1 (5)
C2—C3—C4	120.5 (5)	C1A—C2A—H2A	120.00
C3—C4—C5	120.1 (5)	C3A—C2A—H2A	120.00
Cl4—C4—C3	118.5 (4)	C2A—C3A—H3A	120.00
Cl4—C4—C5	121.5 (4)	C4A—C3A—H3A	120.00
Cl5—C5—C6	118.2 (4)	C4A—C5A—H5A	120.00
C4—C5—C6	120.6 (4)	C6A—C5A—H5A	120.00
Cl5—C5—C4	121.1 (4)	C1A—C6A—H6A	120.00
C1—C6—C5	119.3 (5)	C5A—C6A—H6A	120.00
O11—C11—O12	126.0 (4)	O4A—C41A—H41A	110.00
O11—C11—C1	117.9 (4)	O4A—C41A—H42A	110.00
O12—C11—C1	116.0 (4)	O4A—C41A—H43A	109.00
O21—C21—O22	125.4 (5)	H41A—C41A—H42A	109.00
O21—C21—C2	113.2 (4)	H41A—C41A—H43A	109.00
O22—C21—C2	121.3 (4)	H42A—C41A—H43A	109.00
			
C41A—O4A—C4A—C5A	−1.5 (9)	C2—C3—C4—Cl4	−179.4 (4)
C41A—O4A—C4A—C3A	−178.9 (5)	C2—C3—C4—C5	1.4 (8)
C6—C1—C2—C21	−176.1 (4)	Cl4—C4—C5—C6	180.0 (4)
C6—C1—C2—C3	2.2 (6)	C3—C4—C5—Cl5	−178.8 (4)
C2—C1—C6—C5	−1.6 (7)	Cl4—C4—C5—Cl5	2.0 (7)
C11—C1—C6—C5	178.4 (4)	C3—C4—C5—C6	−0.8 (8)
C2—C1—C11—O11	81.2 (6)	C4—C5—C6—C1	0.9 (8)
C2—C1—C11—O12	−101.9 (5)	Cl5—C5—C6—C1	179.0 (4)
C6—C1—C11—O11	−98.9 (5)	N1A—C1A—C2A—C3A	−177.2 (4)
C11—C1—C2—C3	−177.8 (4)	C6A—C1A—C2A—C3A	1.9 (7)
C11—C1—C2—C21	3.8 (6)	N1A—C1A—C6A—C5A	178.9 (5)
C6—C1—C11—O12	78.0 (5)	C2A—C1A—C6A—C5A	−0.2 (7)
C1—C2—C21—O21	19.1 (6)	C1A—C2A—C3A—C4A	−3.5 (8)
C1—C2—C21—O22	−163.9 (4)	C2A—C3A—C4A—O4A	−179.1 (5)
C21—C2—C3—C4	176.3 (4)	C2A—C3A—C4A—C5A	3.4 (8)
C3—C2—C21—O22	17.8 (6)	O4A—C4A—C5A—C6A	−178.9 (5)
C3—C2—C21—O21	−159.3 (4)	C3A—C4A—C5A—C6A	−1.7 (9)
C1—C2—C3—C4	−2.1 (7)	C4A—C5A—C6A—C1A	0.1 (8)

### Hydrogen-bond geometry (Å, º)

**Table:**

*D*—H···*A*	*D*—H	H···*A*	*D*···*A*	*D*—H···*A*
N1*A*—H11*A*···O11^i^	0.81	2.55	2.926 (5)	110
N1*A*—H11*A*···O22^ii^	0.81	2.10	2.881 (5)	163
N1*A*—H12*A*···O11^iii^	0.87	1.95	2.811 (5)	168
N1*A*—H13*A*···O12	0.99	1.84	2.814 (5)	167
O21—H21···O12^iii^	0.95	1.53	2.480 (4)	179
C3*A*—H3*A*···O12^iv^	0.95	2.50	3.265 (7)	137

Symmetry codes: (i) *x*+1/2, *y*+1/2, *z*; (ii) *x*−1/2, *y*+1/2, *z*; (iii) *x*+1/2, −*y*+1/2, −*z*+1; (iv) *x*+1, *y*, *z*.

## References

[bb1] Agilent (2012). *CrysAlis PRO* Agilent Technologies, Yarnton, England.

[bb2] Altomare, A., Cascarano, G., Giacovazzo, C. & Guagliardi, A. (1993). *J. Appl. Cryst.* **26**, 343–350.

[bb3] Farrugia, L. J. (2012). *J. Appl. Cryst.* **45**, 849–854.

[bb4] Flack, H. D. (1983). *Acta Cryst.* A**39**, 876–881.

[bb5] Mattes, R. & Dorau, A. (1986). *Z. Naturforsch. Chem. Sci.* **41**, 808–814.

[bb6] Odabaşoğlu, M. & Büyükgüngör, O. (2007). *Acta Cryst.* E**63**, o4374–o4375.

[bb7] Sheldrick, G. M. (2008). *Acta Cryst.* A**64**, 112–122. 10.1107/S0108767307043930 18156677

[bb8] Smith, G., Wermuth, U. D. & White, J. M. (2009). *Acta Cryst.* E**65**, o2111. 10.1107/S160053680903044X PMC2970019 21577526

[bb9] Smith, G. & Wermuth, U. D. (2012). *Acta Cryst.* E**68**, o1928. 10.1107/S1600536812023458 PMC3379483 22719681

[bb10] Smith, G., Wermuth, U. D. & White, J. M. (2008*a*). *Acta Cryst.* C**64**, o180–o183. 10.1107/S0108270108004034 18322349

[bb11] Smith, G., Wermuth, U. D. & White, J. M. (2008*b*). *Acta Cryst.* C**64**, o532–o536. 10.1107/S0108270108027054 18758028

[bb12] Spek, A. L. (2009). *Acta Cryst.* D**65**, 148–155. 10.1107/S090744490804362X PMC2631630 19171970

